# Sydney ‘lockout’ liquor licensing law restrictions have been associated with a sustained reduction in emergency department presentations from assaults over 5 years

**DOI:** 10.1111/1742-6723.13955

**Published:** 2022-03-09

**Authors:** James W Deacon, Paul Preisz, Anthony J Chambers

**Affiliations:** ^1^ Department of General Surgery St Vincent's Hospital Sydney Sydney New South Wales Australia; ^2^ Department of Emergency Medicine St Vincent's Hospital Sydney Sydney New South Wales Australia; ^3^ St Vincent's Clinical School The University of New South Wales Sydney New South Wales Australia

**Keywords:** alcohol drinking, emergency medicine, injuries, violence

## Abstract

**Objectives:**

The present study assessed the impact of changes to the New South Wales Liquor Act in 2014 on assault‐related presentations to the ED of St Vincent's Hospital. This hospital is the primary receiving hospital for the area affected by these laws.

**Methods:**

Patients presenting to the ED with an assault‐related diagnosis were identified from the ED and trauma registry databases from 2009 to 2019 and retrospectively reviewed. The number of presentations in the 5 years prior to the introduction of the laws in 2014 was compared to the number occurring in the 5 years following this. Admission to the intensive care unit (ICU) and in‐hospital death were used as markers for severe injury.

**Results:**

From 2009 to 2019 there were 2983 assault‐related presentations to the ED, with 153 requiring ICU admission and 12 deaths. The mean number of presentations annually fell from 342 to 255 after the introduction of the laws (*P* = 0.01). The reduction in presentations was sustained for the entire 5‐year period after the introduction of the laws. Although the mean number of patients requiring ICU admission per year fell from 17 to 14, and the mean number of deaths annually fell from 1.6 to 0.8, neither of these were statistically significant.

**Conclusions:**

There has been a significant reduction in assault‐related presentations to St Vincent's Hospital following the changes to the liquor licensing laws that has been sustained for 5 years with no significant decrease in the those with severe assault injuries.

## Graphical abstract

The present study assessed the impact of changes to liquor licensing laws affecting the central business district and Kings Cross entertainment precinct of Sydney in 2014 on presentations to the ED of St Vincent's Hospital, the primary receiving hospital for these areas. The number of assault‐related presentations during the 5‐years prior to the changes to the laws was compared to those in the 5‐years following their introduction. The mean number of presentations annually fell from 342 to 255 after the introduction of these restrictions (*P* = 0.01).

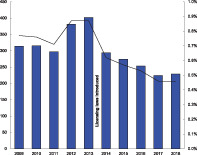

**Key findings**

- Changes to the Sydney liquor licensing laws in 2014 have been associated with a significant reduction in assault‐related presentations to the ED at St Vincent's Hospital.
- The reduction in assault‐related presentations since 2014 has been sustained for 5 years.
- There was no significant reduction in intensive care unit admissions or mortality following assault after changes to these laws.

## Introduction

Assault‐related injuries pose a significant public health concern in Australia. In New South Wales, assault is the third most common cause for ED major trauma admissions, after falls and road trauma.^1^ The excessive consumption of alcohol is also a significant public health issue in Australia and New Zealand, being implicated in almost one in 10 presentations to an ED.^2^ Excessive alcohol consumption can also be a factor in incidents of interpersonal violence and assault.^3^

Governments in multiple jurisdictions in Australia, New Zealand and other countries have implemented a range of different regulatory measures in efforts to reduce the incidence of alcohol‐related assaults and interpersonal violence in the community.^4^, ^5^, ^6^ These have included restrictions on the opening hours of licensed venues serving alcohol, restricting the times where entry and re‐entry of patrons is permitted, limiting the times that alcohol can be served within these premises, restricting the types of alcoholic beverages available for sale in these venues, and restricting the trading hours of off‐licence businesses selling alcoholic beverages.^4^, ^5^, ^6^

In 2008, New South Wales' second largest city Newcastle implemented restrictions on the opening hours of licensed venues and the sale of alcohol ‘shots’ that was associated with a 34% reduction in the number of assaults in that city over a period of 18 months, with a sustained reduction in these incidents observed for 5 years.^7^, ^8^

The City of Ballarat (Australia) in 2003 introduced ‘lockout’ restrictions preventing patrons from entering licensed premises after 03.00 hours. A study of the effects of these restrictions on ED assault‐related presentations found that an initial reduction in these cases was short‐lived, subsequently rising to exceed previous levels within 2 years of the introduction of these restrictions.^9^

A number of violent assaults on young males in the inner‐city areas of Sydney in 2013 where alcohol was a factor resulted in significant media attention and public calls for action.^10^ As a result, the New South Wales State Government legislated an amendment of the Liquor Act in February 2014, popularly referred to as the ‘lockout laws.’^11^ This restricted the sale and service of alcohol in licensed premises within the central business district and Kings Cross entertainment precincts of Sydney. Specifically, these laws prohibited the entry of patrons after 01.30 hours, prohibited service of alcohol after 03.00 hours, and banned the sale of alcohol by off‐licence retail stores after 22.00 hours.

The impact of these laws on the night time economy of the City of Sydney was controversial, with some community and industry groups opposing the restrictions on the basis of a perceived negative impact on local businesses and the culture of night time entertainment in the affected areas.^12^ Calls from these groups to relax or repeal the restrictions led the New South Wales Parliament to conduct a formal inquiry into these restrictions in 2019.^13^ As a result of the Joint Parliamentary Committee's findings the State Government significantly relaxed the licensing restrictions in 2020, and repealed them in 2021.^14^, ^15^

St Vincent's Hospital is located within the area affected by these restrictions and is the primary receiving hospital and trauma centre for this area of Sydney. Previous studies from our institution have shown a significant reduction in the number of presentations with alcohol‐related serious injury, trauma and orbital fractures in the 12 months following the introduction of these restrictions.^16^, ^17^

The aim of the present study was to assess the longer‐term impact of the amendments to the New South Wales Liquor Act of 2014 on assault‐related presentations to our ED, and to ascertain whether the observed initial reduction has been sustained. A secondary aim was to assess what impact these restrictions have had on the number of patients with severe injuries from assault, classified as those requiring intensive care unit (ICU) admission or associated with in‐hospital mortality.

## Methods

Patients presenting to the ED of St Vincent's Hospital during the 10‐year period from February 2009 to February 2019 were identified using both the ED information system database and the hospital trauma registry, both of which are prospectively collected.

The ED database was searched to identify patients assigned the following three IDC10 codes: alleged assault (T74.9), head injury (S06.8), and maxillo‐facial injury (S02.4). It should be noted that at our institution patients are only assigned a single ICD code for their presentation in the ED database. Patients with head or maxillofacial injuries that were not sustained in an alleged assault, and patients with injuries from domestic violence incidents, were excluded. Where patients re‐presented to the ED following a recent admission they were only counted once. Patient demographic details, the day of the week of the presentation, and the details of any ICU admission and inpatient deaths were all recorded.

All patients with assault‐related injuries recorded in these databases were included in the analysis. We did not attempt to exclude patients injured in assaults where alcohol may not have been a factor. The purpose of this was to assess the broader public health benefits associated with the introduction of the restrictions, as well as recognising the difficulty in establishing whether alcohol was a factor (affecting either the victim or the perpetrator) in a violent assault from a retrospective review of medical records.

Assault‐related presentations as a percentage of total ED presentations for each year of the study were calculated. For comparison purposes, patients were divided into two groups based on their date of presentation: the 5 years prior to the introduction of the restrictions (February 2009–February 2014) and the 5 years following this (February 2014–February 2019). The number of assault‐related presentations per year, assault‐related presentations as a proportion of total ED presentations, the number of presentations from 00.01 hours Monday through to 23.59 hours Thursday, the number of presentations from 00.01 hours Friday through to 23.59 hours Sunday, and the number of patients requiring ICU admission and in‐hospital deaths occurring per year in these two groups was compared using unpaired two‐tailed Student's *t*‐test assuming unequal variances. The proportion of patients who were male in the two groups was compared using the χ^2^‐test.

The study was approved by the St Vincent's Health Network Human Research Ethics Committee.

## Results

**Figure 1:**
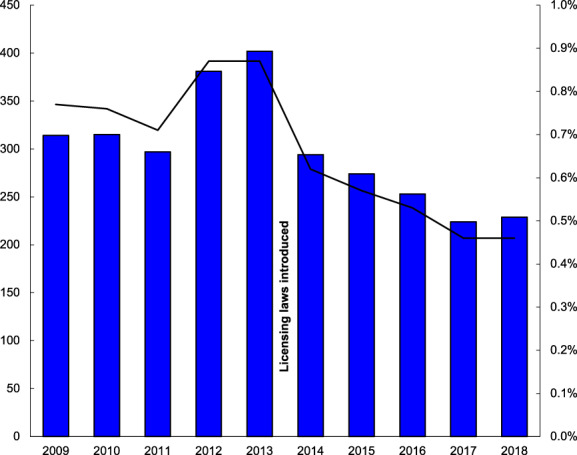
The number of assault‐related ED presentations per year and the percentage of these to total presentations from February 2009 to February 2019 at St Vincent's Hospital. Number of assault‐related presentations for each year (bars) uses the left Y‐axis. Assault‐related presentations per year as a percentage of total ED presentations (line) uses the right Y‐axis.

From February 2009 to February 2019 there were 2983 presentations to the ED of St Vincent's Hospital following an alleged assault. The median age of patients presenting after assault was 30 (range 16–93, interquartile range 24–41). There were 2358 male patients (79%) and 625 female (21%). A total of 153 patients required admission to the ICU (5.1%) and there were 12 in‐hospital deaths occurring (0.4% mortality). During this 10‐year period there was a total of 456 423 presentations to this department, with assault‐related cases representing 0.65% of all presentations. The number of assault‐related presentations occurring per year and the percentage of these to total ED presentations is shown in Figure 1.

In the 5 years prior to the introduction of the licensing law restrictions from February 2009 to February 2014 there was a total of 1706 assault‐related presentations to the ED, with 83 requiring ICU admission and eight deaths. This compares to the 5 years following the introduction of these restrictions from February 2014 to February 2019 where there were 1274 assault‐related presentations, representing an overall reduction of 25%. This included 70 ICU admissions and four in‐hospital deaths.

**Figure 2:**
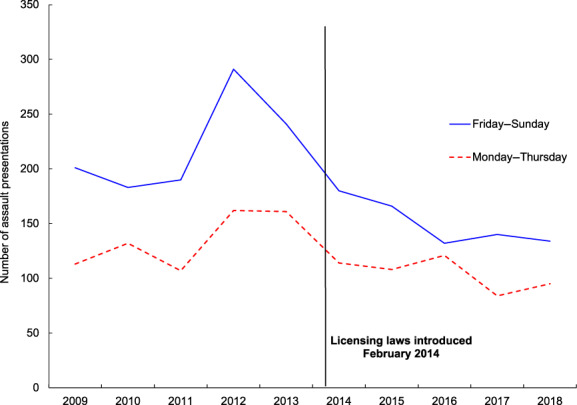
The number of assault‐related ED presentations per year at St Vincent's Hospital from February 2009 to February 2019 showing the number of patients presenting on Mondays through to Thursdays (dashed line) and those presenting on Fridays through to Sundays (solid line).

Table 1 shows a comparison of the demographic details of patients presenting with assault, the number of assault‐related presentations per year, the number of assaults occurring on Mondays through to Thursdays, the number of assaults occurring on Fridays through to Sundays, assault‐related presentations as a percentage of total ED presentations, and the number of patients requiring ICU admission or dying in hospital per year in the 5 years prior to the licensing law restrictions compared to the 5 years following. The number of assault‐related presentations per year presenting on Mondays through to Thursdays and presenting on Fridays through to Sundays is shown in Figure 2.

**TABLE 1:** Assault‐related ED presentations at St Vincent's Hospital in the 5 years prior to the introduction of the liquor licensing laws compared to the 5 years following this

	2009–2014	2014–2019	*P*
Median age (IQR)	29 (23–39)	32 (24–44)	0.008
Percentage male	81%	76%	0.0003
Assault‐related ED presentations per year, mean (SD)	342 (47)	255 (30)	0.01
Assault‐related ED presentations Monday to Thursday per year, mean (SD)	135 (26)	104 (15)	0.05
Assault‐related ED presentations Friday to Sunday per year, mean (SD)	221 (45)	150 (21)	0.02
Assault‐related ED presentations per year as a percentage of total presentations, mean (SD)	0.79% (0.072)	0.53% (0.070)	0.0004
Assault‐related ICU admissions per year, mean	17	14	0.39
Assault‐related inpatient deaths per year, mean	1.6	0.8	0.18

IQR, interquartile range; SD standard deviation.

## Discussion

The present study has shown that since changes to the New South Wales Liquor Act were made in 2014 to restrict the service of alcohol in licensed venues within the central business district and Kings Cross entertainment precinct of Sydney, there has been a significant reduction in the number of assault‐related ED presentations to the primary receiving hospital for this area.

In the 12 months following the introduction of these restrictions there was an immediate fall in the annual number of presentations, confirming the results of a previous study from our institution that showed a significant fall in presentations with alcohol‐related serious injury and trauma for this period.^16^ Our study has shown that this reduction in assault‐related ED presentations has been sustained over a 5‐year period.

Assault‐related presentations on Fridays, Saturdays and Sundays fell to a greater extent than those presenting on Mondays to Thursdays after these restrictions were introduced. This could be explained by fewer assaults occurring within or in proximity to the licensed venues affected by these restrictions that are most popular on Friday evenings and weekends, and that the existing culture of alcohol consumption during these times was to some extent curtailed by the licensing restrictions. We observed small but significant differences in the age and sex of assault‐related presentations to the ED after the introduction of the restrictions, likely reflecting the fact that any assaults prevented by these restrictions would have involved victims that were more likely to be male and of a younger age group.

The sustained reduction in the number of assault‐related ED presentations following the introduction of the licensing law restrictions demonstrated in the present study reflects a benefit to public health outcomes from fewer injuries from alcohol‐related assault, and also in allowing limited ED and hospital resources to be redirected for the treatment of other patients. There is also a significant economic saving to the community in reducing the incidence of these assaults, with the total costs of a single alcohol‐related assault estimated to be more than $85 000 (Australian dollars).^18^

Our findings are consistent with studies of policing statistics that have shown a reduction in the number of assaults reported to police in the areas affected by the licensing law restrictions since these were introduced. Studies from the New South Wales Bureau of Crime Statistics and Research have found a 13.3% reduction in non‐domestic assaults in the area affected by the restrictions, including a 53% decline within the Kings Cross entertainment precinct.^19^ Using this data, Kypri and Livingston calculated that 627 assaults had been prevented over 5 years, which approximates our own findings that there were 432 fewer assault‐related ED presentations in the 5 years after these restrictions compared with the preceding 5 years.^20^

The reduction in assault‐related presentations observed in the present study cannot be explained by changes to the total volume of presentations to our ED as assault‐related cases as a proportion of total ED presentations showed an even greater degree of reduction in the period studied. This was due to an increase in overall ED presentations during this period, and suggests that the incidence of violent assaults leading to injury requiring ED treatment in the area of Sydney that our hospital services has fallen.

The suggestion that reductions in the number of assaults occurring in the area affected by the licensing restrictions can be explained by displacement of alcohol‐related violent incidents into other areas of Sydney remains unsettled. A study by Kypri and Livingston found that although there was a small increase in assaults reported to police in adjacent areas of Sydney following the restrictions, this was more than compensated for by a far larger reduction in assaults occurring in the affected area.^20^ A report from Royal Prince Alfred Hospital, the closest major trauma centre to our institution, by Dinh and colleagues showed no increase in assault‐related trauma presentations in the 2 years following the introduction of the restrictions.^21^

The liquor licensing restrictions were introduced by the New South Wales State Government in response to the deaths of a number of young males in the central business district and Kings Cross entertainment precinct.^10^ The impact of these restrictions on the number of patients presenting with severe injuries requiring ICU admission or dying from their injuries at our institution was less marked than the reduction seen in less seriously injured patients. Although the mean number of patients requiring ICU admission following an assault per year reduced from 17 to 14 in the 5 years following the restrictions compared with the preceding 5 years, and the mean number of patients who died from their injuries per year also reduced from 1.6 to 0.8, neither of these reductions reached statistical significance. Our study suggests that the liquor licensing restrictions have had a greater impact on reducing ED presentations with less serious injuries from assaults than on those with more severe or life‐threatening injuries from these incidents. The reasons for this observation are unclear; however, it is possible that assaults occurring within or in proximity to licensed venues may be associated with a lower severity of injury than assaults occurring in other contexts that were less likely to be impacted by the restrictions. It is also possible that the degree of intoxication with alcohol of either the victim or the perpetrator of an assault may influence the severity of the injuries sustained, and hence may have been affected by the licensing restrictions.

The liquor licensing laws, although generally accepted to have been successful in reducing the incidence of alcohol‐related violence in inner‐city Sydney, have also been controversial for the effects that they have had on the night time economy of these precincts and the businesses they contain.^12^ In response to these concerns the Parliament of New South Wales conducted a review of the restrictions, leading the Government to relax the restrictions in 2020, and eventually to repeal them in 2021.^13^, ^14^, ^15^ Restrictions on individuals and businesses in the City of Sydney during the COVID‐19 pandemic in 2020 and 2021 during periods of ‘lock‐down’ has meant that presentations to our ED following assault are currently at historically low levels despite the repeal of the licensing law restrictions. It is our intention to continue to track the number of assault‐related presentations to our institution in future years to assess what impact the lifting of these restrictions may have.

Limitations of the present study include its retrospective nature and the use of our ED database that only records a single ICD code for each presentation. This may have underestimated the actual number of assault‐related presentations if these were assigned a code for an injury other than those for assault, head injury or maxillofacial injury. As we used the same inclusion criteria for the entire period studied, the results and trends identified would still be valid. Another limitation of the present study is the lack of a control group in another location outside of the area affected by these restrictions for comparison. This limits the ability of the present study to account for other factors that may have impacted assault‐related presentations other than the licensing law restrictions. Such factors may include police operations and more effective policing, the influence of community advertising campaigns, changes in the price or taxation of alcohol containing beverages, and demographic changes within the area of our study.

## Conclusions

In the 5 years following the introduction of the ‘lockout laws’ amendment of the New South Wales Liquor Act in 2014 there has been a significant and sustained reduction in assault‐related presentations to the ED of St Vincent's Hospital, the primary receiving hospital of the area affected by these restrictions. Although there was also a reduction in the number of seriously injured patients requiring admission to the ICU or dying from their injuries, this did not reach statistical significance. Further studies should examine the impact that the repeal of these restrictions in 2021 may have on presentations with assault in future years.

## Data Availability

The data that support the findings of this study are available from the corresponding author upon reasonable request.
